# Internet gaming disorder and psychological well-being among university students in Egypt

**DOI:** 10.1186/s40359-023-01418-6

**Published:** 2023-11-03

**Authors:** Aya Shouman, Warda Abo Elez, Ibtihal M.A. Ibrahim, Mohammed Elwasify

**Affiliations:** Department of psychiatry, Faculty of medicine, Mansoura university Hospitals, Mansoura University, Mansoura, Egypt

**Keywords:** Gaming disorder, University students, Psychological well-being, Internet gaming, Addictive behavior

## Abstract

**Background:**

Internet gaming disorder (IGD) is a serious rising problem affecting people of all ages. Many researchers reported that students’ addictive gaming behavior resulted in the loss of function and the development of psychological problems. In this study, we aimed mainly to measure the prevalence of internet gaming disorder among Mansoura University students and find its relationship with psychological well-being.

**Methods:**

A cross-sectional observational study was carried out during the academic year (2021–2022) at the University of Mansoura. Students from four different faculties were included. Participants ages ranged from 18 to 25 years old. An online Google Form questionnaire gathering the tools (questionnaire of demographic and clinical data, Internet Gaming Disorder Short Form scale, Ryff’s scale of psychological well-being) was distributed among them.

**Results:**

In this study, 870 students were included. The age range was 18–25 years. They were divided into three groups: 315 normal gamers (36%), 500 risky gamers (58%), and 55 disordered gamers (6%), with no significant gender difference in each group (p-value = 0.138). A negative correlation was found between IGD and psychological well-being (r = -0.303).

**Conclusions:**

The prevalence of IGD was 6% among Mansoura University students. Participants in the theoretical faculties who started playing internet gaming at a younger age and spent more than 2 h per week playing and more than 3 h per week thinking about playing internet games were more likely to develop IGD. Whenever IGD scores increased, psychological well-being scores were found to decrease (r = -0.303).

## Background

The definition of “addiction” has evolved throughout time. The word is derived from the Latin word “addicere,“ which means “bound to” or “enslaved by,“ and when it was first used, it was not exclusively related to drug usage [1].

Addiction is defined by the following characteristics: continued engagement in a behavior despite harmful consequences; a lack of self-restraint when engaging in the behavior; the urge and craving prior to such involvement; and compulsive involvement. Given that these are the defining characteristics of addictive behavior, an addiction framework may be applicable to behaviors other than those associated with substance use [1], such as street drugs, nicotine, and some inappropriately taken prescription medications [2].

Moreover, behavioral addiction refers to frequent behaviors that are beyond normal frequency and increasingly create a dependence that impacts a person’s life [3]. The core element of behavioral addiction was described by **Kardefelt-Winther et al. (2017)** as the persistent and sustained functional impairment that results directly from the addicted behavior [4].

Internet addiction, also known as problematic internet usage (PIU) in the literature, is growing more commonplace on a global scale. According to international research, around half of the teenagers suffer negative effects from excessive internet use [5].

The validity of the majority of studies as an indicator of public health is challenged by the fact that participants’ individual internet usage has not been consistently defined. Because internet usage can include a wide range of activities such as gaming, information gathering, online shopping, networking, gambling, and sex, additional research may show that each of these activities is a separate entity disorder or a subtype of IA [5].Researchers have focused more on the Internet gaming problem since the first commercial video game was created in the early 1970s.especially after a number of violent incidents linked to gaming-related problems [6].

Results showed that behavioral addiction or impulse-control disorders could be used to interpret the unfavorable consequences of online gaming. Researchers were able to identify the negative effects of digital gaming and explore its nature, prevalence, and pathogenesis with the help of the criteria for pathological gambling or substance dependency [7].

Internet gaming disorder (IGD) was added to the 5th edition of the Diagnostic and Statistical Manual of Mental Disorders (DSM-5) in Section III, Under Conditions for Further Study, in 2013. It was defined as the repeated and persistent use of internet games that results in clinically significant impairment or distress [8]. Gaming disorder (GD) was included in the International Classification of Diseases (ICD-11) as an addictive disorder in 2018 [9].

Even though playing video games is generally safe and can have useful physical, cognitive, and social effects [10], excessive gaming has been linked to a number of negative effects, such as sleep disruptions, solitude, relationship issues, job loss, inadequate nutrition, and fitness, in addition to grief, isolation, decreased intellectual activity, and dissatisfaction with physical appearance [11; 7].

### Aim of the work

In this study, we aimed to measure the prevalence of internet gaming disorder among Mansoura University students and find whether there was a relationship between it and the psychological well-being of the affected students.

## Methods

This is a cross-sectional observational study that was carried out during the academic year 2021–2022 (from December 2021 to February 2022) at the University of Mansoura. A total of 870 students from four different faculties—medicine, engineering, arts, and education (two practical and two theoretical faculties)—were included. Participants ages ranged from 18 to 25 years old; institutional review board (IRB) approval was obtained prior to the study (MS.21.05.1502). A Pilot study was carried out among 30 students at different faculties before the actual field work to assess the feasibility of our research design and it was more reliable to carry out the study through an Online google Form. An online Google Form questionnaire gathering the tools described below (questionnaire of demographic and clinical data, Internet Gaming Disorder Short Form scale, Ryff’s scale of psychological well-being) was delivered via an online link and distributed among the students. And all participating students agreed to participate in the study at the beginning of the form and were asked if they play Internet games (yes/no). By answering “yes,” they were included in the survey and by answering “no,” they were excluded.

### Study questionnaires

**Demographic variables**; A structured questionnaire containing sociodemographic data, basic psychiatric and medical data, and information about the pattern of internet and gaming use and most activity of interest on the internet.

**A validated Arabic version of the IGD short form scale (IGD SF);** [12] which is based on the nine criteria from the DSM-5, was used. Analysis of the short dichotomous scale indicated that three groups could be differentiated as follows: normal gamers (scores between 0 and 2), risky gamers (scores ranged between 1 and 6), and disordered gamers (scores ranged between 6 and 9) [13].

**The Arabic version has 42 items. Ryff’s scale of psychological well-being (SPWB);** The scale was translated by Jondi and Talahmeh (2017) [14]. This scale comprises six subscales: autonomy, environmental mastery, purpose in life, personal growth, positive relations with others, and self-acceptance. Each subscale is composed of 7 items. Contributors respond to one of six-point categories ranging from (1) strongly disagree to (6) strongly agree. The scores were in the range of 54–324, with higher scores signifying better psychological well-being.

**Fig. 1:**
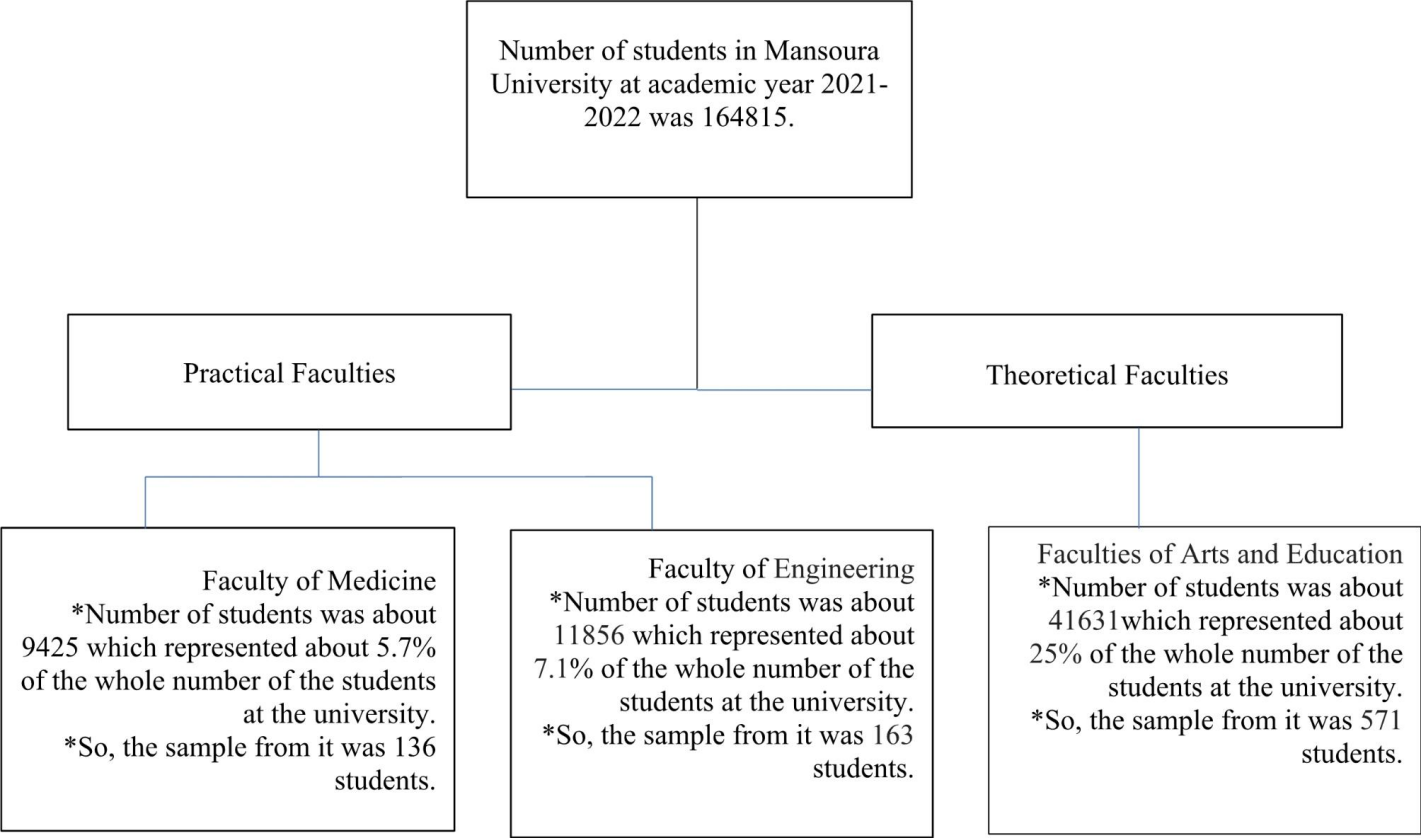
Flowchart of sample size calculation

**Sampling and sample size calculation** Our sample size was calculated using the Open Epi program (http://www.openepi.com/SampleSize/SSPropor.htm). A previous study reported that 25.2% of university students have IGD [15]. A sample size of at least 850 students was required with an alpha error of 5%, a precision of 5%, and a design effect of three due to the stratified cluster sampling method, as illustrated by the flowchart (Fig. 1). Participants had to be Mansoura University students of Egyptian nationality in order to be considered. Students with psychotic disorders were excluded.

During a period of 3 months (from 1st of December 2021 to 28th February 2022), one of the researchers obtained approval from the head of each faculty in order to communicate personally with the study subjects in the selected faculties to explain study objectives and encourage participation. Then, the distribution of the questionnaire was done by sharing Google links to WhatsApp or Facebook personal accounts or WhatsApp or Facebook groups of the mentioned faculties. The snowball sampling technique (exponential, non-discriminative) was applied by encouraging participants to share the link with other students in their faculties. The responses were collected, and the link was locked when the target sample size was reached. All study participants were assured that the data collected would be kept confidential and anonymous, and they agreed to participate in the study at the beginning of the Google form.

### Statistical analysis

Data analysis was performed by SPSS software, version 18 (SPSS Inc., PASW Statistics for Windows version 18). Chicago: SPSS Inc. Numbers and percentages were used to describe qualitative data. After assessing normality with the Kolmogorov-Smirnov test, quantitative data were reported using the median (minimum and maximum) for non-normally distributed data and the mean and standard deviation for regularly distributed data. The Chi-Square and Monte Carlo tests were used to compare qualitative data. For normally distributed data, the Student test was used to compare two independent groups; for more than two groups, the one-way ANOVA test with the post hoc Tukey test to identify pairwise comparisons was used. The observed results’ significance was determined at the 0.05 level.

## Results

**Sociodemographic characteristics of the studied group**, 870 students filled in the questionnaire. Their mean age was 20.83 years ± 1.73, they consisted of 462 males (53.1%) and 408 females (46.9%), 490 of them were living in rural areas (56.3%) while 380 were living in urban areas (43.7%). Data were collected from 4 different faculties (Engineering, Medicine, Education and Arts) from 6 different classes. (Table 1**)**.

**Table 1:** sociodemographic data of the study group:

	N = 870	%
Age/years		
mean ± SD	20.83 ± 1.73	
Sex		
Male Female	462 408	53.1 46.9
Residence		
Rural Urban	490 380	56.3 43.7
Faculty		
practical theoretical	299 571	34.4 65.6
Marital status		
Not engaged Engaged	37 833	4.3 95.7
Academic year		
1st 2nd 3rd 4th 5th 6th	114 107 329 235 40 45	13.1 12.3 37.8 27.0 4.6 5.2
Number of studying hours (hour/day)		
< 2 2 < 4 4 < 6 ≥6	171 345 283 71	19.7 39.7 32.5 8.2

**Regarding internet use**, 283 (32.5%) students spent more than 6 h per day on the internet, and of them, 24 (3.7%) reported using it mainly for playing games. In terms of starting age, primary school (207), preparatory school (232), and secondary school (219) **(**Table 2**).**

**Table 2:** Pattern of internet and gaming use

	N = 870	%
Time spent in front of screen (hours)		
< 1 h 2–3 4–5 ≥ 6	64 231 292 283	7.4 26.6 33.6 32.5
Most activity of interest on the internet	N = 642	
Watching films Using social media Communication with friends Information seeking Playing games Others	50 343 142 47 24 36	7.8 53.4 22.1 7.3 3.7 5.6
Age of starting gaming		
Never KG Primary Preparatory Secondary	193 19 207 232 219	22.2 2.2 23.8 26.7 25.2
Duration of playing (hour/week)		
Median (min-max)	2(0.5–78)	
Time passed thinking of playing on internet (hour/week)		
Median (min-max)	3(0.25-19)	

h: hour, *Others (listening to music/podcast, uploading/downloading contents, sending or reading E-mails, distant learning)

**Fig. 2:**
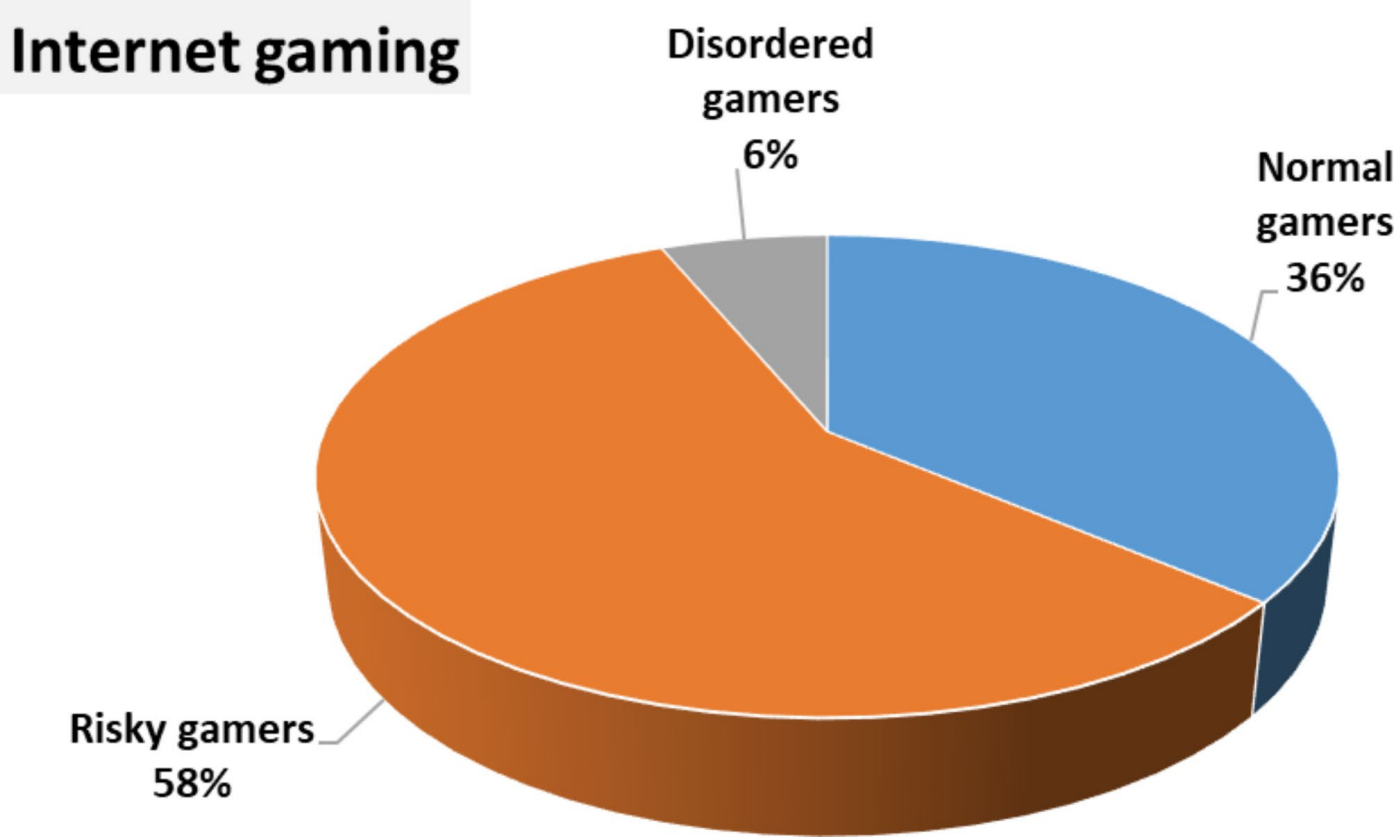
Distribution of internet gaming scale

**Regarding the distribution of internet gaming**, there were 500 students considered to be risky gamers, 55 disordered gamers, and 315 normal gamers. (Fig. 2)

**Internet gaming in relation to gender**, 27 male students were disordered gamers versus 28 females while 280 males were risky gamers vs. 220 females thus results show a non-statistically significant difference (p value = 0.138) between males and females regarding Internet gaming disorder.

**Univariate analysis of the predictors of risky and disordered gamers among studied students**, the risk of internet gaming disorder was found to be increased in students of theoretical faculties (faculties of education and arts) with high odds (1.4 more times at risk than practical faculties), students of the 1st year of faculty with higher odds of 5.78 times at risk than students of subsequent years, and students who spent more than 4 h in front of a screen (2.08 times at risk). Even students who spent more than 6 h in front of a screen were 2.44 times more likely to develop gaming disorder than those who played more than 2 h per week (3.15 times riskier) or spent more than 3 h per week thinking about playing internet gaming (2.54 times riskier). **(**Table 3**)**

**Table 3:** Univariate analysis of the predictors of risky and disordered gamers among studied students

	Total N = 870	Internet gamers		χ^2^	P value	Odds ratio (95%CI)
		**Normal** **N = 315**	**Risky & disordered gamers** **N = 555**			
Faculty						
Theoretical	**571**	191(33.5)	380(66.5)	5.46	0.019*	1.41(1.06–1.88)
o Arts and education						
Practical( r )						
o Engineering o Medical	**299** 163 136	124(41.5)	175(58.5)			
Academic year						
1st 2nd 3rd 4th 5th 6th (r )	114 107 329 235 40 45	15(13.2) 50(46.7) 134(40.7) 79(33.6) 16(40) 21(46.7)	99(86.8) 57(53.3) 195(59.3) 156(66.4) 24(60) 24(53.3)	20.68 0.0001 0.575 2.80 0.383 R	< 0.001* 0.994 0.448 0.094 0.536 R	5.78(2.59–12.84) 0.998(0.496-2.0) 1.27(0.681–2.38) 1.73(0.906–3.29) 1.31(0.554–3.11) R
Time spent in front of screen						
< 1 (r ) 2–3 4–5 ≥ 6	64 231 292 283	33(51.6) 97(42) 99(33.9) 86(30.4)	31(48.4) 134(58) 193(66.1) 197(69.6)	R 1.86 7.02 10.38	1 0.172 0.008* 0.001*	R 1.47(0.844–2.56) 2.08(1.20–3.58) 2.44(1.40–4.23)
Age of starting gaming						
Never( r )						R
KG Primary Prep Secondary	193 19 207 232 219	147(76.2) 7(36.8) 45(21.7) 49(21.1) 67(30.6)	46(23.8) 12(63.2) 162(78.3) 183(78.9) 52(69.4)	1 13.46 118.54 128.46 13.48	< 0.001* < 0.001* < 0.001* 0.002*	5.47(2.04–14.73) 11.50(7.21–18.36) 11.93(7.56–18.85) 2.48(1.52–4.05)
Duration of playing (hour/week)#						
≤ 2 (r ) > 2	188 181	57(30.3) 22(12.2)	131(69.7) 159(87.8)	18.08	< 0.001*	3.15(1.83–5.42)
Time passed thinking of playing on internet #(hour/week)						
≤ 3 (r ) > 3	323 240	86(26.6) 30(12.5)	237(73.4) 210(87.5)	16.79	< 0.001*	2.54(1.61-4.0)

r: reference group, *statistically significant

χ2 = Chi-Square test, p:probability, Binary logistic regression

**Multivariate analysis of the predictors of risky gamers among studied students**, students of theoretical faculties who started playing at a young age, played more than 2 h per week, and thought about playing for more than 3 h every week were found to have a higher risk of developing internet gaming disorder, and these factors can predict the risk of developing IGD by 82.2%. **(**Table 4**)**

**Table 4:** multivariate analysis of the predictors of risky gamers among the study group:

Predictors of risky gaming	Β	P-value	Adjusted Odds ratio (95%CI)
Age of starting gaming			
Never( r )			1
KG Primary Prep Secondary	1.087 2.24 1.45 1.65	0.285 0.001* 0.02* 0.013*	2.96(0.404–21.77) 9.41(2.42–36.52) 4.29(1.23–14.97) 5.21(1.42–19.14)
Duration of playing (hour/week)# ≤ 2(r ) > 2	0.708	0.028*	2.03(1.08–3.82)
Time passed thinking of playing on internet #(hour/week)			
≤ 3 (r ) > 3	1.06	0.003*	2.88(1.43–5.79)

Overall % predictors = 82.2%

r: reference group, *statistically significant, β; regression co-efficient

### Psychological well-being (SPWB) scale results among the study group

**Fig. 3:**
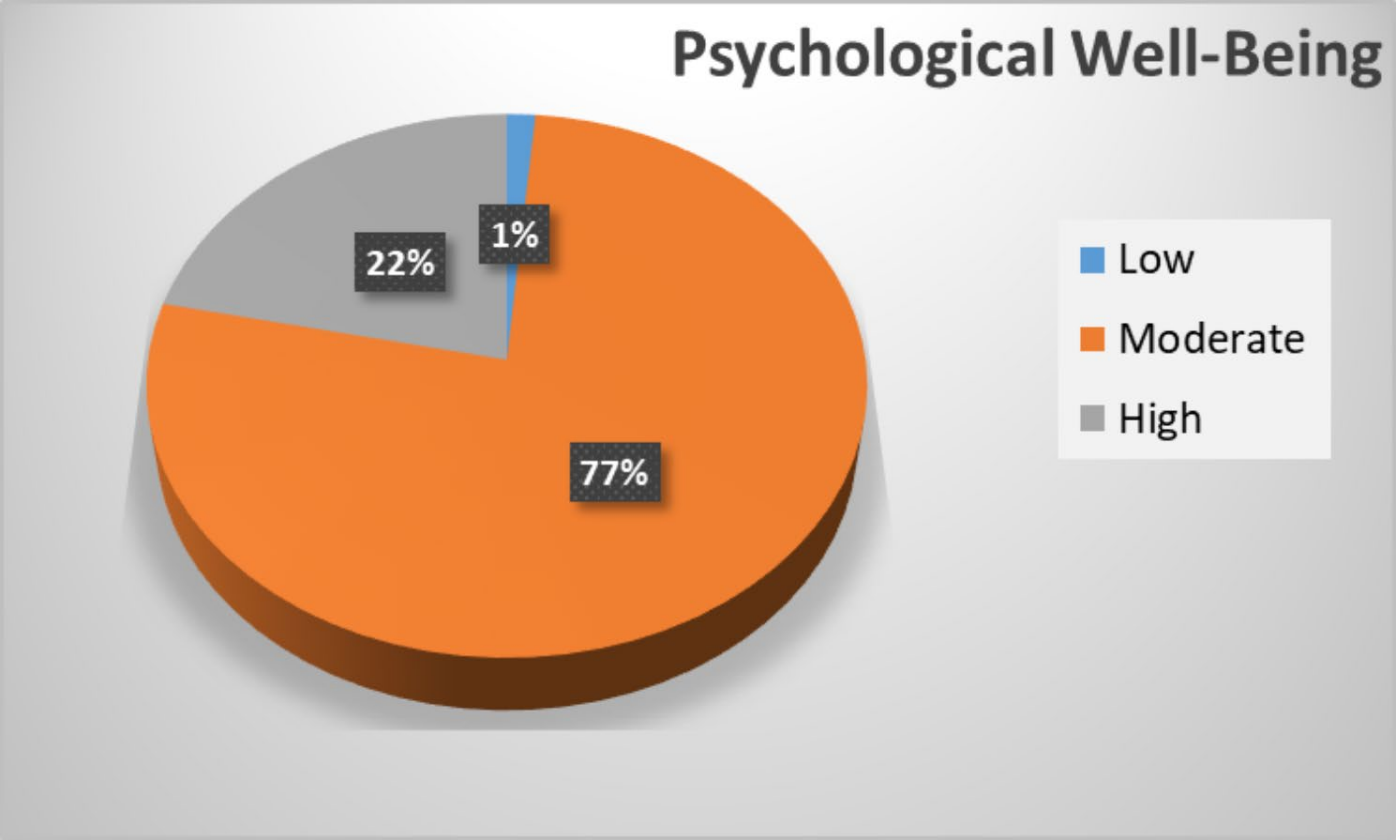
Distribution of psychological well-being scale

The result of **Ryff** Scale of Psychological Well-Being (SPWB) about 670 (77%) students had moderate scores of psychological wellbeing, 188 (21.6%) of them had high scores and only 12 (1.4%) of the total sample had low scores. (Fig. 3)

**Fig. 4:**
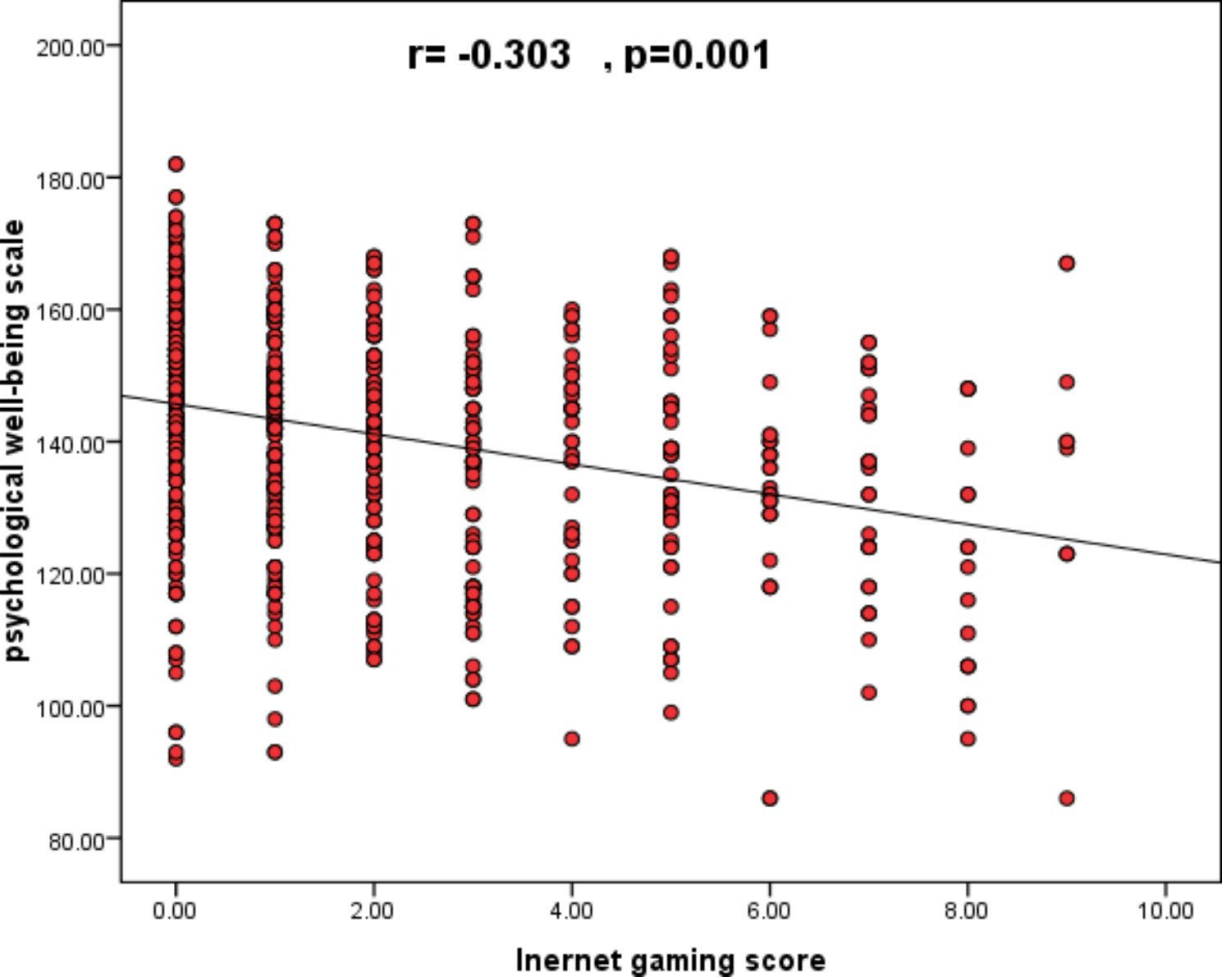
Scatter diagram showing the correlation between internet gaming disorder scores and psychological well-being scale scores among the sample

**The relation between internet gaming disorder (IGD) and psychological well-being among the sample**: an inverse relationship was found between the severity of the internet gaming disorder and the psychological wellbeing. (Fig. 4)

**The relation between psychological well-being & internet gaming among the sample.** It was found that 500 students were risky gamers, and only six of them had low scores of psychological wellbeing, while 402 had moderate scores and 92 had high scores. Disordered gamers were 55 students; only 2 of them had low psychological wellbeing scores, while 49 had moderate scores and 4 had high scores.

**Studying the relationship between internet gaming behavior and each domain of psychological wellbeing**, it was found that all 6 dimensions of psychological well-being were affected in gamers with more affection in risky and disordered gamers than normal ones. **(**Table 5**)**

**Table 5:** relation between Internet gaming and domains of psychological well-being among studied students:

Psychological well-being	Normal gamers N = 315	Risky gamers N = 500	Disordered gamers N = 55	test of significance	
Autonomy	24.80 ± 3.72	23.69 ± 4.21	23.35 ± 5.52	F = 7.89 P < 0.001*	
environmental mastery	23.48 ± 3.40	22.04 ± 3.24	20.71 ± 3.89	F = 25.94 P < 0.001*	
personal growth	24.84 ± 3.76	24.27 ± 3.53	21.63 ± 4.93	F = 17.49 P < 0.001*	
positive relations	24.66 ± 4.31	23.27 ± 4.34	22.04 ± 4.37	F = 14.25 P < 0.001*	
purpose in life	24.22 ± 3.44	22.58 ± 4.24	21.04 ± 4.10	F = 24.43 P < 0.001*	
Self-acceptance	24.98 ± 3.45	23.11 ± 4.42	21.40 ± 4.59	F = 29.03 P < 0.001*	

F: One Way ANOVA test, p: probability, parameters described as mean ± SD *statistically significant

**Linear regression of prediction of psychological well-being among studied group by internet gaming scores** shows statistically significant results assuming internet gaming score to be a negative predictor for psychological well-being with the following equation (Prediction equation of psychological well-being = 145.68–2.276* internet gaming score). **(**Table 6**)**

**Table 6:** linear regression of prediction of psychological well-being among studied cases by internet gaming score

	Β	T	P -value
internet gaming score	-2.276	9.37	< 0.001*

Prediction equation of psychological well-being = 145.68–2.276* internet gaming score

β: correlation co-efficient

## Discussion

In this study, the prevalence of disordered gamers was found to be 6%, while risky gamers were 58%, and normal gamers were 36%. There was no significant difference in gaming behavior between males and females. These results were consistent with previous studies’ results, such as **Yu and Cho (2016)**, who reported that 5.9% of studied subjects were disordered gamers [16]. Furthermore, half of the subjects in an Egyptian study by **Gammal et al. (2019)** were at risk of acquiring Internet Gaming Disorder (IGD) [17].

Moreover, a cross-sectional survey conducted online by **Almutairi et al. (2023)**, on Arab gamers from Syria, Jordan, and Kuwait revealed that the prevalence of disordered gamers across these countries was 6.1%, which supports our result [18].

In contrast, the prevalence of risky gamers was different from that detected by other studies. **Festl et al. (2013)** and **ELNahas et al. (2018)** reported a lower prevalence of risky gamers than what was found in this study [19; 15]. The present increase in the incidence of dysregulated gaming behavior could be attributed to the rise in internet usage and the availability of gaming apps. This increase was particularly noticeable during the coronavirus pandemic in 2019 because of the reliance on online learning and home isolation, which created more free time and increased the sense of loneliness [20]. Another factor contributing to the higher risk of IGD among college students could be the fact that parents might not be able to effectively monitor their kids’ gaming behaviors and attitudes by the time they go to college [21].

In the current research, some predictors of increased risk of internet gaming disorder among the sample were detected, including four main predictors: type of faculty, age at which the student started playing, hours spent playing per week, and hours spent thinking about playing each week.

Participants in theoretical faculties (education and arts) were found to be 1.2 times more at risk for Internet gaming disorder (IGD) than participants in practical faculties (medical and engineering); this may be related to increased studying time and lab work compared to theoretical faculties. Such a result agrees with the results of previous studies where students of theoretical faculties were found to be at higher risk for gaming disorder than those of practical ones [15, 17].

Students started playing at a young age, especially at the age of primary school, when they were 9.41 times riskier than other participants. This result agrees with the results of previous studies [22–24]. However, these findings disagree with the results of a study carried out by **Malak et al. (2017)**, who found that excessive and inappropriate internet use was greater among students who used the Internet during adolescence, particularly after the age of 16 [25]. Also, **Hur (2006)** showed that the age of first internet use was not a risk factor for problematic internet use [26].

Playing online games for longer periods of time during the week increased the risk of IGD. Thus, students who played more than 2 h per week were found to be 2.03 times more at risk than students playing less than 2 h per week; this result agrees with the results of many previous studies [23, 27–29]. Moreover, another study found that the weekly average of time spent playing online positively correlated with the risk of IGD [30]. However, **Billieux et al. (2013)** noted that time dedicated to gaming is not always a reliable indicator of problematic gaming behavior [31].

Students who spent more than 3 h per week preoccupied with and thinking about gaming had a higher risk of developing gaming disorder (2.88 times riskier than gamers who spent less than 3 h per week). This was similar to the results of a previous study, which assumed that preoccupation is one of the most relevant diagnostic criteria of all nine IGD DSM-5 proposed criteria [32].

Other studies disagree with our result; preoccupation was reported at high rates by participants in one study, but it was weak in predicting IGD [33]. Also, **Charlton and Danforth (2007)** distinguished the core as well as the ancillary criteria of behavioral addiction and found that preoccupation was not a sign of addiction but rather a non-pathological engagement factor [34].

In the present study, a negative relationship was found between the severity of the internet gaming disorder and psychological wellbeing. The higher a person’s IGD score, the lower his psychological well-being score. These results were consistent with findings in previous studies, which assumed that loneliness increases the risk of problematic internet use and IGD [35–38].

Also, **Akn and Iskender (2011)** discovered a link between problematic internet use and low self-esteem [39]. Moreover, **Ballou and Van Rooij (2021)** studied the relationship between mental well-being and dysregulated gaming and consistently detected that both were negatively correlated, which further supports our findings in this research [40]. These previously mentioned findings may be explained by the compensatory and the interpersonal impairment hypotheses set for explaining the psychopathology of gaming disorder [41–43].

The outcome in this current study showing that the disordered gamers had decreased scores in purpose in life and self-acceptance domains of psychological well-being, is also in line with previous studies; a cross sectional study proposed that higher levels of IGD are associated with lower perceived life satisfaction and self-acceptance among adolescents and older adults and two longitudinal studies concluded that IGD negatively predicted life satisfaction; one study was carried among adults over 40 years old and the other among younger population with age range 17–21 years [44–46]. These data strongly support the uses and gratifications theory explaining pathological gaming behavior [46].

However, Orben and Przybylski, (2019) carried out research about the association between adolescent well-being and digital technology use and found negative association between digital technology use and adolescent well-being but they mentioned that it was small to be significant [47].

### Limitations

This study has some potential limitations that should be considered when interpreting the findings reported. It is a cross-sectional study, so future longitudinal studies need to be done to identify the nature of the associations between IGD and psychological wellbeing. Scales were self-reported and completed online, leading to psychological biases such as social desirability and memory recall bias. Future research aiming at examining how IGD affects psychosocial well-being should consider combining various data collection methods, such as teacher or parent ratings and expert evaluation. This is an online study with low external validity because the results can’t be generalized. Further research is required to examine IGD among various age groups since our study only looked at one particular age group.

## Conclusions

The prevalence of IGD was 6% and showed no significant gender difference. However, there was an inversely proportionate relationship between gaming behavior and psychological well-being, as when IGD scores increased, psychological well-being scores decreased. Still, it is not clear whether decreased psychological well-being is a cause or effect of internet gaming disorder, and it is recommended to address this issue much more in further research among adolescents and university students. Some predictors for developing gaming disorder in university students were noted; those who were in theoretical faculties, started playing internet gaming at a younger age, and spent too many hours playing and thinking about playing internet games were more likely to develop IGD.

## Data Availability

The data are available upon request.

## List of abbreviations

IGD: Internet Gaming Disorder
IA: Internet addiction
PIU: Problematic internet use
DSM-5: The Diagnostic and Statistical Manual of Mental Disorders fifth edition
ICD-11: The eleventh revision of the International Classification of Diseases
IRB: Institutional review board
IGD SF scale: Internet gaming disorder short form scale
SPWB: Scale of psychological well-being
SPSS: Statistical Package for the Social Sciences
PASW: Predictive Analytics Software (applied statistical software)
ANOVA: Analysis of Variance
COVID-19: Coronavirus Disease 2019
WHO: World Health Organization
